# Isoscattering strings of concatenating graphs and networks

**DOI:** 10.1038/s41598-020-80950-6

**Published:** 2021-01-15

**Authors:** Michał Ławniczak, Adam Sawicki, Małgorzata Białous, Leszek Sirko

**Affiliations:** 1 Institute of Physics, Polish Academy of Sciences, Aleja Lotników 32/46, 02-668 Warsaw, Poland; 2 Center for Theoretical Physics, Polish Academy of Sciences, Aleja Lotników 32/46, 02-668 Warsaw, Poland

**Keywords:** Physics, Quantum physics, Theoretical physics

## Abstract

We identify and investigate isoscattering strings of concatenating quantum graphs possessing *n* units and 2*n* infinite external leads. We give an insight into the principles of designing large graphs and networks for which the isoscattering properties are preserved for $n \rightarrow \infty$. The theoretical predictions are confirmed experimentally using $n=2$ units, four-leads microwave networks. In an experimental and mathematical approach our work goes beyond prior results by demonstrating that using a trace function one can address the unsettled until now problem of whether scattering properties of open complex graphs and networks with many external leads are uniquely connected to their shapes. The application of the trace function reduces the number of required entries to the $2n \times 2n$ scattering matrices ${\hat{S}}$ of the systems to 2*n* diagonal elements, while the old measures of isoscattering require all $(2n)^2$ entries. The studied problem generalizes a famous question of Mark Kac “Can one hear the shape of a drum?”, originally posed in the case of isospectral dissipationless systems, to the case of infinite strings of open graphs and networks.

## Introduction

The celebrated question of Marc Kac “Can one hear the shape of a drum?”^1^ was posed to address the problem of isospectral drums having the same shape. Mathematically, this question is equivalent to the distinctiveness of spectra of the Laplace operator on planar domains with Dirichlet boundary conditions. The negative answer to Marc Kac question was formulated by Gordon, Webb, and Wolpert^2,3^. Using Sunada’s theorem^4^ they constructed different in shape pairs of isospectral dissipationless domains in $\mathbb {R}^{2}$. These important theoretical findings were confirmed experimentally by Sridhar and Kudrolli^5^ and Dhar et al.^6^ who used for this purpose especially designed pairs of microwave isospectral cavities. One should point out that the isospectral properties of pairs of neutrino billiards with the shapes of various isospectral in the nonrelativistic limit billiards have been recently investigated numerically in Ref.^7^. It was found that the isospectrality of the billiards is lost when changing from the nonrelativistic to the relativistic case.

The problem of isospectrality was also analyzed in other important physically, mathematically and technologically quantum systems—quantum graphs. Quantum graphs are the unions of vertices connected by one-dimensional quantum bounds^8,9^. The importance of quantum graphs stems from the fact that they can be used to describe a huge number of physical and mathematical systems and models, e.g., nanophotonic lasers on graphs^10^, superconducting quantum circuits^11^, entanglement in graph states^12,13^, experimental setups for high-dimensional multipartite quantum states^14^, quantum circuits in tunnel junctions^15^, and Weyl and non-Weyl quantum graphs and networks^16^.

Gutkin and Smilansky^17^ proved that the spectrum of a graph can be used to uniquely identify graph’s structure if the lengths of its bonds are incommensurable. However, in the case of graphs with commensurate lengths of bonds the situation is more complicated. Among infinitely many realizations of graphs with the same total length $\mathcal {L}$ it is also possible to find the isospectral dissipationless ones, which are characterized by different topological properties. An effective method of construction of such graphs uses the representation theory and the transplantation technique^18,19^.

In real life open physical systems, including quantum graphs with leads^20^ and microwave networks, one have to deal with dissipation of energy due to, e.g., internal absorption and coupling to the outside world. In such a situation one can ask a more general question whether the geometry of a graph can be revealed in scattering-type experiments. Also in this case the question was answered in negative. To find this answer Band, Sawicki and Smilansky^21,22^ analyzed isospectral quantum graphs with attached two infinite leads. They theoretically demonstrated that among such graphs it is possible to identify the isoscattering ones.

The theoretical findings were experimentally confirmed in the series of papers^23–25^ where a pair of isoscattering quantum graphs with two external infinite leads were simulated by two isoscattering microwave networks. In the experimental analysis relatively simple isoscattering networks were characterized by standard characteristics of isoscattering properties of graphs such as the cumulative phase and the structures of poles of the determinant of the two-port scattering matrices.

In this article we present the construction of isoscattering strings resulting from concatenating open quantum graphs and microwave networks. The strings are constructed from *n* building blocks (units), each one possessing two external leads, and therefore they can be characterized by $2n \times 2n$ scattering matrices ${\hat{S}}$. Using the transplantation technique we prove that the strings are isoscattering, i.e. they have the same spectra of scattering matrices. It means that their scattering matrices can be, for example, characterized by the same cumulative phases of their determinants which is a standard indicator of isoscattering^23–25^. We show that their isoscattering properties are preserved in the limit $n \rightarrow \infty$. Furthermore, we demonstrate that a trace function can be used as a much simpler and therefore a much more effective tool for identifying of complex isoscattering networks. The theoretical predictions are confirmed experimentally for $n=2$, i.e., strings of four-leads microwave networks.

## Isoscattering strings of concatenating graphs

**Figure 1:**
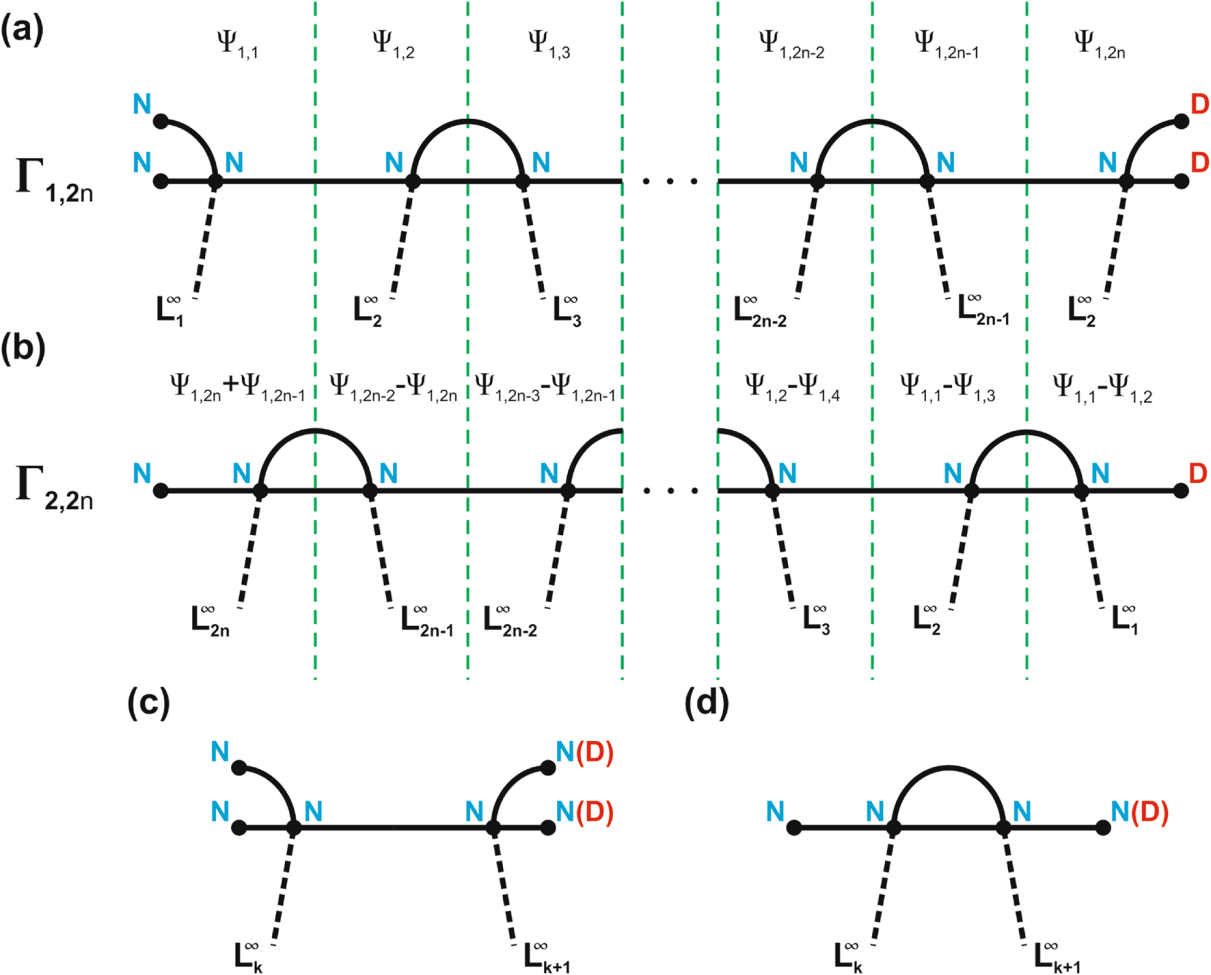
Schemes of the isoscattering strings of the concatenating graphs $\Gamma _{1,2n}$ and $\Gamma _{2,2n}$. (**a**) The isoscattering string $\Gamma _{1,2n}$ with $n-1$ loops, 2*n* leads, and $V=2n+4$ vertices. (**b**) The isoscattering string $\Gamma _{2,2n}$ with *n* loops, 2*n* leads, and $V=2n+2$ vertices. The Neumann and Dirichlet boundary conditions are marked by N and D capital letters, respectively. The restriction of the wave function $\hat{\Psi }_{1,2n}$ to a segment *k* of the string $\Gamma _{1,2n}$ is denoted by $\Psi _{1,k}$. The wave function $\Phi _{2,k}$ restricted to a segment *k* of $\Gamma _{2,2n}$ can be expressed by the components of the wave function $\hat{\Psi }_{1,2n}$ using the formulas (2–3). (**c**–**d**) The elementary units of the isoscattering strings $\Gamma _{1,2n}$ and $\Gamma _{2,2n}$, respectively. The vertices of the internal units possess the Neumann boundary conditions. The vertices of the last units of the strings from the right-hand side fulfil the Dirichlet boundary conditions.

We demonstrate that the two strings of concatenating open graphs $\Gamma _{1,2n}$ and $\Gamma _{2,2n}$ given in Fig. 1a, b are isoscattering. For this we construct the transplantation matrix that transforms a wave function $\hat{\Psi }_{1,2n}$ with the frequency $\nu$ defined on $\Gamma _{1,2n}$ to a wave function $\hat{\Phi }_{2,2n}$ with the same frequency $\nu$ defined on $\Gamma _{2,2n}$. Both $\hat{\Psi }_{1,2n}$ and $\hat{\Phi }_{2,2n}$ satisfy all the vertex conditions of $\Gamma _{1,2n}$ and $\Gamma _{2,2n}$, respectively. The construction goes as follows. We divide each string of graphs into 2*n* building segments (see Fig. 1c, d). The restriction of the wave function $\hat{\Psi }_{1,2n}$ to a segment *k* is denoted by $\Psi _{1,k}$ and similarly the restriction of $\hat{\Phi }_{2,2n}$ to a segment *k* is denoted by $\Phi _{2,k}$. Our goal is to show that there is a $2n\times 2n$ matrix ${\hat{T}}_{2n}$ that is independent of $\nu$ and satisfies:

1 $$\begin{aligned} \begin{pmatrix} \Phi _{2,1} \\ \vdots \\ \Phi _{2,2n} \end{pmatrix}= {\hat{T}}_{2n}\begin{pmatrix} \Psi _{1,1} \\ \vdots \\ \Psi _{1,2n} \end{pmatrix} \end{aligned}$$

One can easily see that if we put

2 $$\begin{aligned} \Phi _{2,2n}& {}= \Psi _{1,2n-1}+\Psi _{1,2n},\,\,\,\,\,\Phi _{2,1}=\Psi _{1,1}-\Psi _{1,2},\, \end{aligned}$$

3 $$\begin{aligned} \Phi _{2,k}& {}= \Psi _{1,k-1}-\Psi _{1,k+1},\,\,\,\, {\text{for}}\, k\in \{2,\ldots ,2n-1\}, \end{aligned}$$

then $\hat{\Phi }_{2,2n}$ satisfies the vertex conditions of $\Gamma _{2,2n}$ provided that $\hat{\Psi }_{1,2n}$ does so on $\Gamma _{1,2n}$. Therefore, the transplantation matrix ${\hat{T}}_{2n}$ is given by:

4 $$\begin{aligned} {\hat{T}}_{2n}=\begin{pmatrix} 1&{}-1&{}0&{}&{}&{}&{}&{}\\ 1&{}0&{}-1&{}0&{}&{}&{}&{}\\ 0&{}1&{}0&{}-1&{}0&{}&{}&{}\\ &{}0&{}1&{}\ddots &{}\ddots &{}\ddots &{}&{}\\ &{}&{}\ddots &{}\ddots &{}\ddots &{}\ddots &{}\ddots &{}\\ &{}&{}&{}0&{}1&{}0&{}-1&{}0\\ &{}&{}&{}&{}0&{}1&{}0&{}-1\\ &{}&{}&{}&{}&{}0&{}1&{}1 \end{pmatrix}. \end{aligned}$$

The existence of transplantation guarantees that the $2n \times 2n$ scattering matrices ${\hat{S}}^{(I)}$ and ${\hat{S}}^{(II)}$ of the open graphs $\Gamma _{1,2n}$ and $\Gamma _{2,2n}$ are conjugated through the matrix ${\hat{T}}_{2n}$,

5 $$\begin{aligned} {\hat{S}}^{(I)} ={\hat{T}}_{2n}^{-1}{\hat{S}}^{(II)} {\hat{T}}_{2n}. \end{aligned}$$

To see this denote by $\hat{f}_{1,2n}$ and $\hat{f}_{2,2n}$ the restrictions of $\hat{\Psi }_{1,2n}$ and $\hat{\Phi }_{2,2n}$ to infinite leads, respectively. We have

6 $$\begin{aligned} \hat{f}_{1,2n}& {}= \overline{a}_{1,{\text{in}}}e^{-ikx}+S^{(I)}\overline{a}_{1,{\text{in}}}e^{ikx}, \end{aligned}$$

7 $$\begin{aligned} \hat{f}_{2,2n}& {}= \overline{a}_{2,{\text {in}}}e^{-ikx}+S^{(II)}\overline{a}_{2,{\text {in}}}e^{ikx}. \end{aligned}$$

Next, note that the transplantation can be restricted to infinite leads. Thus the existence of the transplantation implies

8 $$\begin{aligned}&\overline{a}_{2,{\text {in}}}={\hat{T}}_{2n}\overline{a}_{1,{\text {in}}}, \end{aligned}$$

9 $$\begin{aligned}&S^{(II)}\overline{a}_{2,{\text {in}}}={\hat{T}}_{2n}S^{(I)}\overline{a}_{1,{\text {in}}}. \end{aligned}$$

Combining these two equations we obtain

10 $$\begin{aligned} S^{(II)}{\hat{T}}_{2n}\overline{a}_{1,{\text {in}}}={\hat{T}}_{2n}S^{(I)}\overline{a}_{1,{\text {in}}}, \end{aligned}$$

which is equivalent to (5).

## Isoscattering strings of concatenating microwave networks

The simulation of quantum graphs by microwave networks is attainable because of a direct analogy between the telegraph equation characterizing a microwave network and the Schrödinger equation of the complementary quantum graph^26–28^. Microwave networks allow for the simulation of quantum graphs described by three basic ensembles in the random matrix theory (RMT): the Gaussian orthogonal ensemble (GOE)^16,23,26,29–32^, characterized by time (*T*) invariance, the Gaussian unitary ensemble (GUE)^26–28,33,34^ without *T*-invariance, and the Gaussian symplectic ensemble (GSE)^35^ also characterized by *T*-invariance. Many significant papers on this topic^16,27,29,35,36^ clearly demonstrate that microwave networks are particularly useful in the investigation of properties of open quantum graphs with complex topology. Microwave networks and coupled waveguides can also be used to study a topological edge invariant^37^ and the photon number statistics of coherent light^38^. Recently, microwave networks have been applied to realization of the chiral orthogonal, unitary, and symplectic ensembles^39^.

In the experiment described in this article strings of microwave networks simulating strings of quantum graphs with preserved time invariance symmetry were used. They are composed of microwave joints (vertices) connected by coaxial cables (bonds). Each microwave joint (vertex) *i* of a network is connected to the other joints by $v_{i}$ bonds. The number $v_{i}$ is defined as the valency of the joint *i*. In the construction of strings of networks the SMA-RG402 microwave coaxial cables were used. They consist of two conductors: the inner one of radius $r_1$ which is surrounded by an outer conductor of radius $r_2$. A material with the dielectric constant $\varepsilon =2.06$ is used to fill the space between the conductors. Below the onset of the TE$_{11}$ mode, $\nu _{TE} \simeq \frac{c}{\pi (r_1+r_2) \sqrt{\varepsilon }} \simeq 33$ GHz^40^, where *c* denotes the speed of light in the vacuum, inside a coaxial cable is satisfied the condition for propagating only the TEM mode.

**Figure 2:**
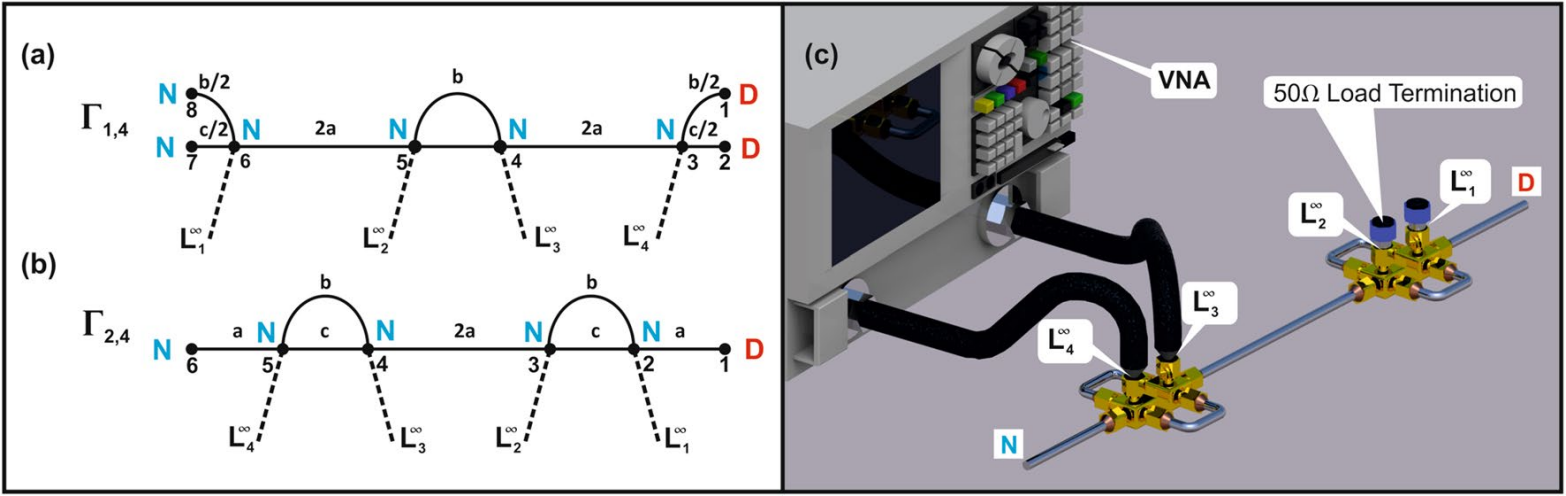
The schemes of the isoscattering strings of concatenating graphs $\Gamma _{1,4}$ and $\Gamma _{2,4}$ with $n=2$ units and 4 leads. (**a**) The isoscattering string of graphs $\Gamma _{1,4}$ with 4 leads $L_1^{\infty },\ldots , L_4^{\infty }$ and $V=8$ vertices. (**b**) The isoscattering string of graphs $\Gamma _{2,4}$ with 4 leads $L_1^{\infty },\ldots , L_4^{\infty }$ and $V=6$ vertices. The Neumann and Dirichlet boundary conditions are marked by N and D capital letters, respectively. (**c**) The experimental realization of the string of concatenating graphs $\Gamma _{2,4}$. The microwave test port cables of the VNA are connected to the vertices 5 and 4 in order to measure the diagonal elements $S^{(II)}_{44}$ and $S^{(II)}_{33}$ of the scattering matrix ${\hat{S}}^{(II)}$. The connection of the VNA to a microwave network is equivalent to attaching of two infinite leads $L^{\infty }_4$ and $L^{\infty }_3$ to a quantum graph. To the vertices No. 3 and 2, 50 Ω loads were attached as the realization of the two additional leads $L^{\infty }_2$ and $L^{\infty }_1$.

The pair of the isoscattering strings of concatenating networks $\Gamma _{1,4}$ and $\Gamma _{2,4}$ obtained from the two elementary units shown in Fig. 1c, d, respectively, are demonstrated in Fig. 2a, b. In Fig. 2c we show the scheme of the isoscattering strings of concatenating microwave networks $\Gamma _{2,4}$ used in the experiment.

For the above strings of graphs and networks the two most typical vertex boundary conditions will be considered, the Neumann and Dirichlet ones. The Neumann boundary condition enforces the continuity of waves meeting at the joint (vertex) *i* and vanishing of the sum of their derivatives at *i*. The Dirichlet boundary condition requires vanishing of the waves at a given joint.

The string $\Gamma _{1,4}$ of the $n=2$ graphs in Fig. 2a consists of $V=8$ vertices connected by $B=8$ bonds. The valency of the vertices $3-6$ is $v=4$ while for the other ones the valency is $v=1$. The vertices with numbers $1-2$ satisfy the Dirichlet boundary conditions, while for the vertices $3-8$ the Neumann boundary conditions are satisfied. The second string $\Gamma _{2,4}$, shown in Fig. 2b, consists of $V=6$ vertices connected by $B=7$ bonds. At the vertex 1 the Dirichlet boundary condition is imposed while the vertices with the numbers $2-6$ satisfy the Neumann boundary conditions.

The strings of concatenating microwave networks $\Gamma _{1,4}$ and $\Gamma _{2,4}$ have the following optical lengths of the bonds:

$$\begin{aligned} b/2& {}= 0.0537 \pm 0.0005 {{\text{ m,}}} \\ c/2& {}= 0.0508 \pm 0.0005 {\text{ m,}} \\ a& {}= 0.1597 \pm 0.0005 {\text{ m,}} \\ b& {}= 0.1074 \pm 0.0005 {\text{ m,}} \\ c& {}= 0.1016 \pm 0.0005 {\text{ m,}} \\ 2a& {}= 0.3194 \pm 0.0005 {\text{ m.}} \\ \end{aligned}$$

In contrast to the investigated in^23^ systems which consisted of simple isoscattering networks with $L=2$ external leads (one unit, $n=1$, of the considered in this article strings) here we study experimentally much more complex isoscattering microwave strings of concatenating networks, consisting of $n=2$ units and having $L=4$ external leads. Because of larger number of leads the systems are more open to the external world. The strings of the networks $\Gamma _{1,4}$ and $\Gamma _{2,4}$ are described by $2n \times 2n$ scattering matrices ${\hat{S}}^{(I)}$ and ${\hat{S}}^{(II)}$, respectively. The relationship between both matrices is given by

11 $$\begin{aligned} {\hat{S}}^{(I)} ={\hat{T}}_4^{-1}{\hat{S}}^{(II)} {\hat{T}}_4, \end{aligned}$$

where ${\hat{T}}_4$ is $4\times 4$ transplantation matrix (4). In general, the application of a standard measure of isoscattering such as the phase of scattering matrix determinant

12 $$\begin{aligned} {\mathrm{Im}}\Bigl [\log \Bigl (\det \bigl ({\hat{S}}^{(I)}\bigr )\Bigr )\Bigr ]= {\mathrm{Im}}\Bigl [\log \Bigl (\det \bigl ({\hat{S}}^{(II)}\bigr )\Bigr )\Bigr ]\text{, } \end{aligned}$$

is from the experimental point of view very inconvenient since for each $2n \times 2n$ scattering matrix ${\hat{S}}^{(I)}$ or ${\hat{S}}^{(II)}$ it requires measurements of $(2n)^2$ matrix elements. If we even assume the reciprocity of the matrices ${\hat{S}}^{(I)}$ and ${\hat{S}}^{(II)}$ for *T*-invariant systems, which imposes that the transmission between any of two “ports” of the matrices does not depend upon the propagation direction, it still requires $2n^2+n$ matrix elements for each of them.

Therefore, we introduce a new measure of isocattering which is the trace of scattering matrices ${\hat{S}}^{(I)}$ and ${\hat{S}}^{(II)}$. Using the properties of the trace function from the formula (5) one obtains

13 $$\begin{aligned} {\text{tr}}{\hat{S}}^{(I)} = {\text{tr}}{\hat{S}}^{(II)}. \end{aligned}$$

One should point out that both functions ${\text{tr}}{\hat{S}}^{(I)}$ and ${\text{tr}}{\hat{S}}^{(II)}$ are complex and depend on microwave frequency $\nu$. The application of the trace function significantly simplifies the experimental procedure. Now, the measurement of only 2*n* diagonal elements of each scattering matrix in a function of frequency $\nu$ is required.

The measurements of the scattering matrices ${\hat{S}}^{(I)}$ and ${\hat{S}}^{(II)}$ were performed using the vector network analyzer (VNA) Agilent E8364B. The experimental procedure is explained in the case of the string of microwave networks $\Gamma _{2,4}$ presented in Fig. 2c.

To measure the diagonal elements $S^{(II)}_{33}$ and $S^{(II)}_{44}$ of the scattering matrix ${\hat{S}}^{(II)}$ the flexible 50 Ω test port cables HP 85133-60016 and HP 85133-60017 of the VNA were connected to the vertices 4 and 5 of the string of microwave networks shown in Fig. 2c. To the vertices 3 and 2, 50 Ω loads were attached as the realization of the two additional leads $L^{\infty }_2$ and $L^{\infty }_1$. The connection of the VNA to a string of microwave networks (see Fig. 2c) is equivalent to attaching of two infinite leads $L^{\infty }_3$ and $L^{\infty }_4$ to a string of quantum graphs. The diagonal elements $S^{(II)}_{11}$ and $S^{(II)}_{22}$ were measured similarly. In this case the VNA was connected to the vertices 2 and 3, while to the vertices 4 and 5, 50 $\Omega$ loads were connected.

The full scattering matrices ${\hat{S}}^{(I)}$ and ${\hat{S}}^{(II)}$, including the off-diagonal elements, are required for testing of the properties of the isoscattering strings using the transplantation matrix ${\hat{T}}_4$. In this case six combinations of connections of microwave test port cables to each string are possible. If any of two vertices of the string were connected to the VNA the remaining two vertices were terminated with 50 $\Omega$ loads.

## Experimental results

**Figure 3:**
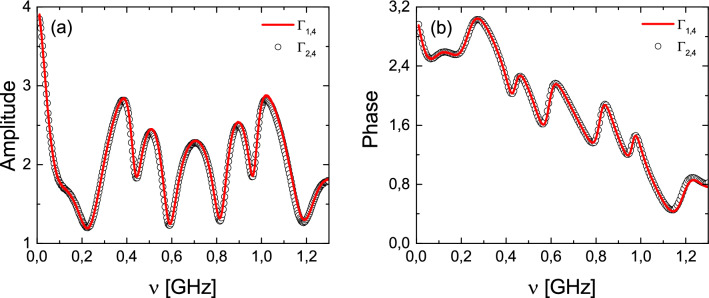
The amplitudes $|{\text{tr}}{\hat{S}}^{(I)}|$ and $|{\text{tr}}{\hat{S}}^{(II)}|$ (**a**) and the phases ${\text{Im}}\Bigl [\log \bigl ({{{\text{tr}}}}{\hat{S}}^{(I)}\bigr )\Bigr ]$ and ${\text{Im}}\Bigl [\log \bigl ({\text{tr}} {\hat{S}}^{(II)}\bigr )\Bigr ]$ (**b**) of the trace function of the scattering matrices ${\hat{S}}^{(I)}$ and ${\hat{S}}^{(II)}$ obtained for the isoscattering strings of concatenating microwave networks $\Gamma _{1,4}$ with $V=8$ vertices (red solid line) and $\Gamma _{2,4}$ with $V=6$ vertices (black empty circles), respectively.

The measurements of the diagonal elements of the scattering matrices ${\hat{S}}^{(I)}$ and ${\hat{S}}^{(II)}$ were performed in the frequency range $\nu$ = 0.01–1.3 GHz. In Fig. 3a we show that the amplitudes of the trace function $|{\text{tr}}{\hat{S}}^{(I)}|$ and $|{\text{tr}}{\hat{S}}^{(II)}|$ of the scattering matrices ${\hat{S}}^{(I)}$ and ${\hat{S}}^{(II)}$ of the strings of networks $\Gamma _{1,4}$ and $\Gamma _{2,4}$, marked by red solid line and black open circles, respectively, are close to each other, proving that we are dealing with the isoscattering networks. For the frequency range 0.01–1 GHz the agreement between the results obtained for both strings of networks is almost perfect. However, for the frequency range 1–1.3 GHz small discrepancies arise, caused possibly by small differences in the cables’ lengths and a small differentiation of the vertex boundary conditions of the networks in a function of frequency $\nu$.

The modulus of the trace function of the scattering matrices can be treated as a concise measure of the isocattering properties of the strings of networks and graphs with dissipation. The problem of losses in microwave networks are discussed in^24^. However, the formula (13) deals with the full trace function which is a complex number. Therefore, the isoscattering properties of the two strings of networks should be also observed in the phases of the trace functions, regardless of the absorption strength

14 $$\begin{aligned} {\text{Im}}\Bigl [\log \bigl ({\text{tr}}{\hat{S}}^{(I)}\bigr )\Bigr ]= {\text{Im}}\Bigl [\log \bigl ({\text{tr}}{\hat{S}}^{(II)}\bigr )\Bigr ]\text{. } \end{aligned}$$

In Fig. 3b we present the comparison of the phases ${\text {Im}}\Bigl [\log \bigl ({\text{tr}}{\hat{S}}^{(I)}\bigr )\Bigr ]$ (red solid line) and ${\text {Im}}\Bigl [\log \bigl ({\text{tr}}{\hat{S}}^{(II)}\bigr )\Bigr ]$ (black empty circles) of the trace function of the scattering matrices ${\hat{S}}^{(I)}$ and ${\hat{S}}^{(II)}$, respectively. The agreement between the results obtained for different networks $\Gamma _{1,4}$ and $\Gamma _{2,4}$ is very good, demonstrating that we deal with the isoscattering strings of networks.

It is important to note that the isoscattering strings of graphs considered in this article have an additional important property, namely the scattering matrices of them are conjugated to each other by the transplantation relation (11), where

15 $$\begin{aligned} {\hat{T}}_{4}=\begin{pmatrix} 1&{}-1&{}0&{}0\\ 1&{}0&{}-1&{}0\\ 0&{}1&{}0&{}-1\\ 0&{}0&{}1&{}1 \end{pmatrix}. \end{aligned}$$

The matrix ${\hat{T}}_4$ does not depend on the frequency and the Eq. (11) is valid for all values of $\nu$.

**Figure 4:**
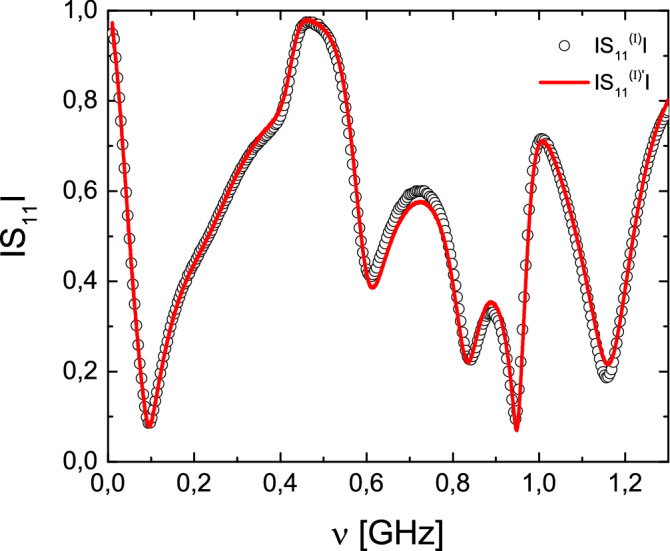
Validation of the transplantation relation (11). The modulus $|S_{11}^{(I)'}|$ (red solid line) that was obtained using the transplantation relation applied to the experimentally measured scattering matrix of the second string $\Gamma _{2,4}$, ${\hat{S}}^{(I)'}={\hat{T}}_4^{-1}{\hat{S}}^{(II)}{\hat{T}}_4$, is compared to the measured modulus of the scattering matrix element $|S_{11}^{(I)}|$ of the first string $\Gamma _{1,4}$ (black empty circles).

In order to check the transplantation relation (11) we transformed experimentally measured scattering matrix of the first string of networks ${\hat{S}}^{(I)'}={\hat{T}}_4^{-1}{\hat{S}}^{(II)}{\hat{T}}_4$ and compared it to the scattering matrix ${\hat{S}}^{(I)}$ of the second one. In Fig. 4 we present the results obtained for the moduli $|S_{11}^{(I)'}|$ (red solid line) and $|S_{11}^{(I)}|$ (black empty circles), respectively. Figure 4 shows clearly that the transplantation relation works very well. Also in this case some small differences that are seen for $\nu > 0.55$ GHz can be attributed to small differences in the cables’ lengths and a small differentiation of the vertex boundary conditions in a function of microwave frequency. In general, however, the transformed scattering matrix of the first string of networks ${\hat{T}}_4^{-1}{\hat{S}}^{(II)}{\hat{T}}_4$ reconstructs very well the scattering matrix of the second one ${\hat{S}}^{(I)}$.

In summary, we proved that there are isoscattering strings of concatenating graphs possessing *n* units and 2*n* infinite external leads. The isoscattering properties of such strings of open graphs are preserved for $n \rightarrow \infty$. The theoretical predictions were confirmed experimentally using the strings of two microwave networks $\Gamma _{1,4}$ and $\Gamma _{2,4}$ consisting $n=2$ units and four-leads which are characterized by the scattering matrices ${\hat{S}}^{(I)}$ and ${\hat{S}}^{(II)}$, respectively. We proved that both systems are isoscattering showing that both matrices are linked by the transplantation relation (11). Furthermore, in the analysis of the strings of microwave networks we used a new measure of isoscattering—the trace function. We demonstrated that the amplitudes $|{\text{tr}}{\hat{S}}^{(I)}|$ and $|{\text{tr}}{\hat{S}}^{(II)}|$, and the phases ${\text {Im}}\Bigl [\log \bigl ({\text{tr}}{\hat{S}}^{(I)}\bigr )\Bigr ]$ and ${\text {Im}}\Bigl [\log \bigl ({{{\text{tr}}}}{\hat{S}}^{(II)}\bigr )\Bigr ]$ of the trace function measured in the frequency range $\nu$ = 0.01–1.3 GHz are very close to each other, proving that both networks are isoscattering.

The application of this new measure reduces the number of required entries of the $2n \times 2n$ scattering matrices ${\hat{S}}$ of the systems to only 2*n* diagonal elements. The measures of isoscattering used in the earlier investigations^23,25^ required all $(2n)^2$ entries of the scattering matrices ${\hat{S}}$. Thus for large open experimental systems, with many leads, they have no operational meaning. The obtained results clearly show that the investigated strings of microwave networks $\Gamma _{1,4}$ and $\Gamma _{2,4}$ are isoscattering paving the way towards the future experimental analysis of even more complex isoscattering systems for which the transplantation relation can be applied. Moreover, after deleting infinite leads, the considered strings of graphs are isospectral as closed quantum systems. They, therefore constitute an interesting example of arbitrary large isospectral quantum graphs.

## Data Availability

The data that support results presented in this paper and other findings of this study are available from the corresponding authors upon reasonable request.
